# Reducing kidney uptake of radiolabelled exendin-4 using variants of the renally cleavable linker MVK

**DOI:** 10.1186/s41181-023-00206-2

**Published:** 2023-09-04

**Authors:** Belinda Trachsel, Giulia Valpreda, Alexandra Lutz, Roger Schibli, Linjing Mu, Martin Béhé

**Affiliations:** 1 Center for Radiopharmaceutical Sciences, ETH-PSI-USZ, Paul Scherrer Institute (PSI), 5232 Villigen, Switzerland; 2 Department of Chemistry and Applied Biosciences, Institute of Pharmaceutical Sciences, ETH Zurich, 8093 Zurich, Switzerland

**Keywords:** Exendin-4, MVK, Nephrotoxicity, Cleavable linkers, Insulinoma imaging, Kidney uptake

## Abstract

**Background:**

Peptidic radiotracers are preferentially excreted through the kidneys, which often results in high persistent renal retention of radioactivity, limiting or even preventing therapeutic clinical translation of these radiotracers. Exendin-4, which targets the glucagon-like-peptide 1 receptor (GLP-1R) overexpressed in insulinomas and in congenital hyperinsulinism, is an example thereof. The use of the tripeptide MVK, which is readily cleaved between methionine and valine by neprilysin at the renal brush border membrane, already showed promising results in reducing kidney uptake as reported in the literature. Based on our previous findings we were interested how linker variants with multiple copies of the MV-motive influence renal washout of radiolabelled exendin-4.

**Results:**

Three exendin-4 derivatives, carrying either one MVK, a MV-MVK or a MVK-MVK linker were synthesized and compared to a reference compound lacking a cleavable linker. In vivo results of a biodistribution in GLP-1R overexpressing tumour bearing mice at 24 h post-injection demonstrated a significant reduction (at least 57%) of renal retention of all ^111^In-labeled exendin-4 compounds equipped with a cleavable linker compared to the reference compound. While the insertion of the single linker MVK led to a reduction in kidney uptake of 70%, the dual approach with the linker MV-MVK slightly, but not significantly enhanced this effect, with 77% reduction in kidney uptake compared to the reference. In vitro IC_50_ and cell uptake studies were conducted and demonstrated that though the cleavable linkers negatively influenced the affinity towards the GLP-1R, cell uptake remained largely unaffected, except for the MV-MVK cleavable linker conjugate, which displayed lower cell uptake than the other compounds. Importantly, the tumour uptake in the biodistribution study was not significantly affected with 2.9, 2.5, 3.2 and 1.5% iA/g for radiolabelled Ex4, MVK-Ex4, MV-MVK-Ex4 and MVK-MVK-Ex4, respectively.

**Conclusion:**

Cleavable linkers are highly efficient in reducing the radioactivity burden in the kidney. Though the dual linker approach using the instillation of MV-MVK or MVK-MVK between exendin-4 and the radiometal chelator did not significantly outperform the single cleavable linker MVK, further structural optimization or the combination of different cleavable linkers could be a stepping stone in reducing radiation-induced nephrotoxicity.

**Supplementary Information:**

The online version contains supplementary material available at 10.1186/s41181-023-00206-2.

## Background

Exendin-4 (Ex4) is an FDA approved incretin mimetic drug clinically available for glycaemic control of diabetes. By agonistic action on the glucagon-like peptide-1 receptor (GLP-1R) expressed on β-cells in the pancreas, Ex4 stimulates the secretion of insulin and reverses states of hyperglycaemia (Goke et al. 1993). Apart from diabetes, the GLP-1R is also an auspicious structural target for the detection of insulinomas (Christ et al. 2009) and congenital hyperinsulinism (CHI) (Boss et al. 2022), which are both conditions of hyper-insulin production resulting in hypoglycaemia. For their diagnosis as well as for an approximation of β-cell mass in diabetes, the use of radiolabelled Ex4 has been widely investigated with promising results (Jansen et al. 2019). In tumour models of pancreatic insulinomas expressing the GLP-1R, Ex4 radiolabelled with the γ-emitter indium-111 accumulated with 287% iA/g in the tumour at 4 h p.i. (Wild et al. 2006). Despite the favourable targeting properties of Ex4, the high unspecific kidney uptake likely mediated by the megalin-cubulin system in the renal brush border membrane (Gotthardt et al. 2007; Vegt et al. 2011), hampers clinical translation. High radiation dose to the kidney can lead to nephrotoxicity after several cycles of peptide receptor radionuclide therapy (PRRT), but also limits the diagnostic value, as due to spatial proximity between the kidneys and the pancreas, GLP-1R positive loci in the tail of the pancreas can be masked and lead to false-negative readings (Antwi et al. 2020). Therefore, reduction of the kidney uptake would not only be desirable in a therapeutic approach, but would facilitate the identification of GLP-1R positive loci in the proximity of the kidney in a diagnostic setting.

**Fig. 1:**
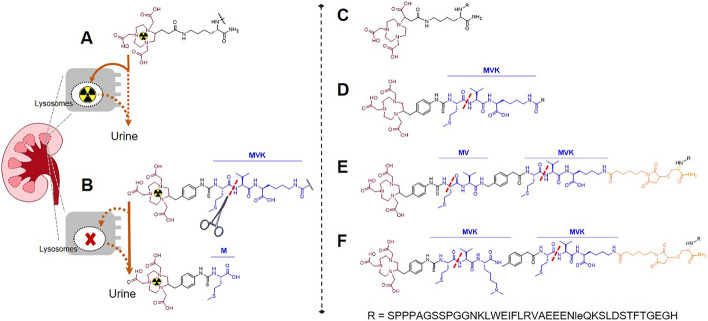
Left: (**A**) Radiolabelled peptides such as [^111^In]In-Ex4 are generally internalized at the kidney brush border membrane via endocytosis and consequently degraded in the lysosomes. The radioisotope together with the chelator persists in the lysosomes, resulting in high radioactivity levels in the kidney. (**B**) The cleavable linker MVK interspaced between the NOTA-chelator and the peptide is cleaved at the Met-Val bond by neprilysin at the apical brush border, generating the hydrophilic radiometal-chelator-Met metabolite, which is readily excreted through the urine. Using this approach, the radioisotope circumvents internalization and lysosomal degradation, alleviating radiation burden to the kidney. Right: Overview of the studied Ex4 derivatives. (**C**) Nle14,Lys40(NODAGA)-Ex4; the reference compound referred to as Ex4; (**D**) Nle14,Cys40(NOTA-Bn-MVK)-Ex4, referred to as MVK-Ex4 containing one cleavable linker sequence; (**E**) Nle14,Cys40(NOTA-Bn-MV-amBn-MVK)-Ex4, referred to as MV-MVK-Ex4 containing two cleavable linker sequences, of which one is comprised of the Lys; (**F**) Nle14,Cys40(NOTA-Bn-MVK(Me)_2_-amBn-MVK)-Ex4, referred to as MVK-MVK-Ex4 containing two full cleavable linker sequences

Consequently, a lot of research has focused on the development of Ex4 derivatives displaying lower kidney uptake. Harnessing the high abundance of albumin in plasma, Käppeli et al. (2019) as well as Ikuni et al. (2022) introduced albumin binding moieties at different sites of the Ex4 sequence, which resulted in a reduced kidney uptake by up to 68% and increased tumour uptake at 4 h p.i. Introduction of cleavable linkers specific to meprin-β, a metalloproteinase highly abundant in the brush border membrane of the kidney, yielded promising in vitro results, but no in vivo reduction of kidney uptake was observed (Jodal et al. 2015). Still, the cleavable linker strategy shows great potential for clinical translation, as it has only a minor influence on pharmacokinetics and does not induce side effects like other kidney reduction strategies such as albumin binders or co-infusion of basic amino acids, respectively (Vegt et al. 2008). Using the novel tripeptidic cleavable linker Met-Val-Lys (MVK) originally conjugated between the 1,4,7-Triazacyclononane-1,4,7-triacetic acid (NOTA) chelator and antibody Fab fragments (Uehara et al. 2018), Zhang et al. (2019) successfully decreased the renal uptake of ^68^Ga-labelled Ex4 by up to 61% at 1 h p.i. The mechanism behind this striking reduction lies in the preferential cleavage of Met-Val bonds by the kidney brush border membrane enzyme neprilysin. Cleavage of this peptide bond generates a hydrophilic metabolite consisting of the radiometal-chelator complex, the complexed radionuclide and a methionine (Met), which is quickly excreted through the urine (Fig. 1A, B). In fact, apart from MVK, which is the most frequently employed cleavable linker in conjunction with radiotracers, a big arsenal of other linking sequences have been described including amongst others: MI (Uehara et al. 2018, 2014), MWK and MFK (Zhang et al. 2021), GYK (Murce et al. 2023) and GFK (Suzuki et al. 2018).

Previously, we have refined Zhang et al.’s approach by instillation of a second MVK motive or an additional MV sequence resulting in the dual cleavable linkers MVK-MVK or MV-MVK, respectively, on the fibronectin-binding peptide FnBPA5.1, which is also suffering from high renal retention of radioactivity (Valpreda et al. 2022). By doubling the MV cleavable site at the brush border membrane, we aimed to augment the availability of the substrate and thereby the probability of cleavage to finally enhance the washout of the radiolabelled peptide from the kidneys. Indeed, in conjunction with FnBPA5.1, the double cleavable linker MV-MVK led to a significantly enhanced washout of radioactivity from the kidneys compared to the single cleavable linker MVK (Valpreda et al. 2022). Therefore, for the current study we wanted to explore if doubling of the cleavage site will also lead to a faster washout of radiolabelled Ex4 and whether it is effective in reducing its dose-limiting kidney retention. Correspondingly, three Ex4 derivatives carrying either one MVK, a MV-MVK or a MVK-MVK cleavable linker sequence were synthesized and tested in vitro and in vivo and were compared to the reference compound Ex4 (Fig. 1C–F). In contrast to Zhang et al. (2019), who used Cys^40^-Leu^14^-Ex4 for their studies, we employed norleucine instead of leucine for substitution of methionine (Cys^40^-Nle^14^-Ex4). Nle^14^ is commonly used as a substitute for Met^14^ to prevent oxidation and has been established for many years.

## Results

### Plasma stability

**Fig. 2:**
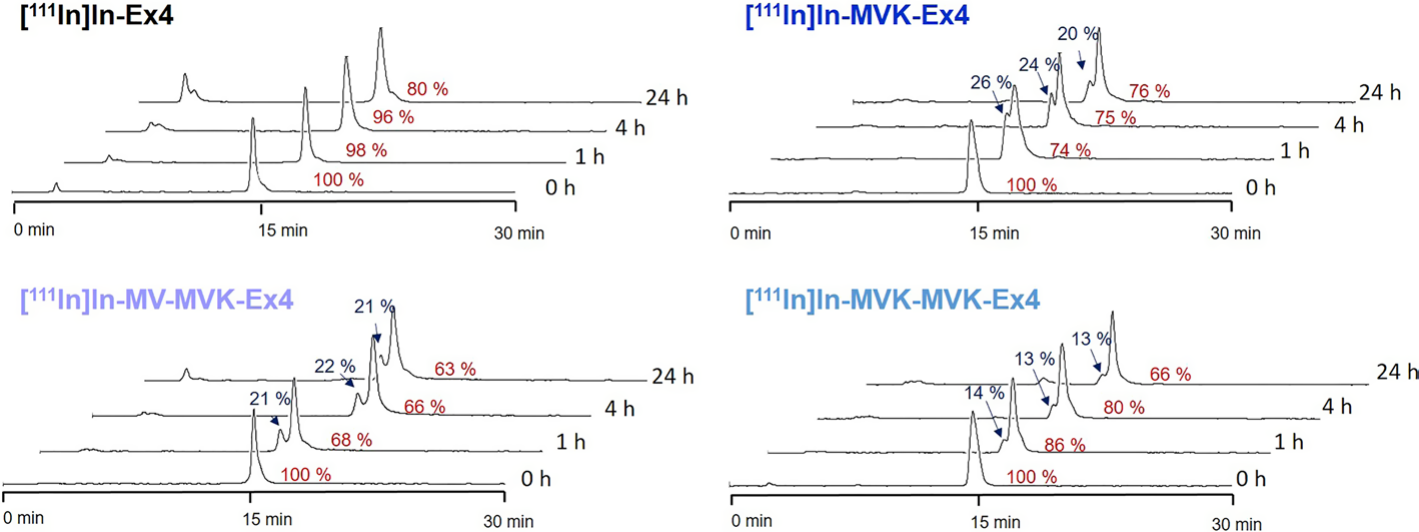
RP-HPLC chromatograms of [^111^In]In-Ex4, [^111^In]In-MVK-Ex4, [^111^In]In-MV-MVK-Ex4 and [^111^In]In-MVK-MVK-Ex4 at different times after incubation with human plasma. In red, stability given as percentage of intact peptide, while the fraction of the secondary peak (blue) is shown next to the arrow. The reference compound Ex4 shows high plasma stability over 4 h with over 96% Ex4 intact, with a consequent drop to 80% intact radiotracer after 24 h. In contrast, Ex4 derivatives equipped with MVK display only 63–76% intact tracer after 24 h and quickly show the generation of an unidentified secondary peak after 1 h exposure to plasma

Ex4 (HGEGTFTSDLSKQNleEEEAVRLFIEWLKNGGPSSGAPPPS-NH2) derivatives MVK-Ex4, MV-MVK-Ex4 and MVK-MVK-Ex4 were labelled with indium-111 and subjected to plasma stability studies. While Ex4 was 80% intact after 24 h incubation in human plasma at 37 °C, all Ex4 derivatives equipped with a cleavable linker displayed lower plasma stabilities with 76%, 63% and 66% intact radiotracer for [^111^In]In- MVK-Ex4, [^111^In]In- MV-MVK-Ex4 and [^111^In]In-MVK-MVK-Ex4, respectively (Fig. 2). Furthermore, all derivatives containing an MVK linker, exhibited the generation of an unidentified secondary peak next to the intact peptide in HPLC chromatograms. This peak could stem from enzymatic breakdown of amino acids distal to the radionuclide, the oxidation of Met, stereochemical changes induced through insertion of the linkers and activated through plasma incubation or as a result of binding to a plasma component.

### Cell uptake and IC_50_

**Fig. 3:**
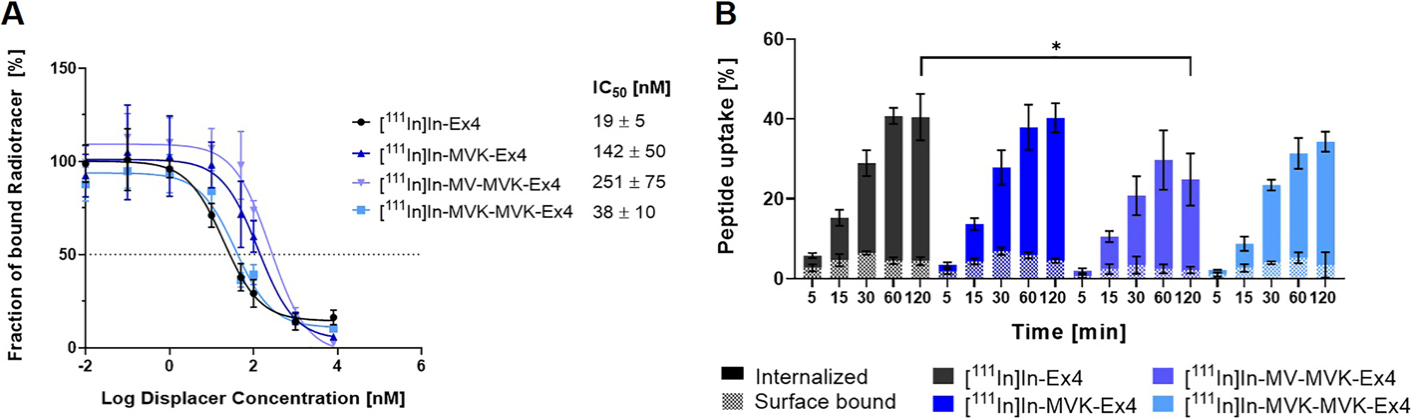
(**A**) Curves depicting the IC_50_ values of [^111^In]In-Ex4 and its derivatives in CHL cells expressing the GLP-1R. The average IC_50_ values are displayed separately in the upper right-hand corner. Significant differences were calculated using the Welch ANOVA test with α < 0.05, which showed that the IC_50_ of all Ex4 derivatives is significantly higher than the IC_50_ of the reference compound [^111^In]In-Ex4. (**B**) Cell uptake and surface binding of radiolabelled Ex4 and its derivatives in CHL cells expressing the GLP-1R after 5, 15, 30, 60 and 120 min. One-way ANOVA comparing [^111^In]In-Ex4 derivatives to the reference compound [^111^In]In-Ex4 was conducted for the percentage of cell uptake at 120 min. Only [^111^In]In-MV-MVK-Ex4 showed significantly lower uptake than [^111^In]In-Ex4 after 120 min. *p < 0.05

Insertion of the cleavable linker sequences MVK, MV-MVK and MVK-MVK resulted in significantly higher IC_50_ values compared to the reference compound [^111^In]In-Ex4. Specifically, [^111^In]In-MVK-MVK-Ex4 displayed the lowest yet significantly increased IC_50_ value of 38 ± 10 nM compared to 19 ± 5 nM of [^111^In]In-Ex4. Meanwhile, both [^111^In]In-MVK-Ex4 and [^111^In]In-MV-MVK-Ex4 exhibited significantly higher IC_50_ values of 142 ± 50 nM and 251 ± 75 nM, respectively (Fig. 3A). Interestingly, the loss in affinity of the Ex4 derivatives did not correspond to a reduced cell uptake, with the exception of [^111^In]In-MV-MVK-Ex4, which displayed significantly lower uptake into the cell at 120 min compared to [^111^In]In-Ex4. All other compounds achieved around 40% cell uptake after 120 min and displayed a constant surface bound-fraction of 5–10% (Fig. 3B).

### Biodistribution

**Fig. 4:**
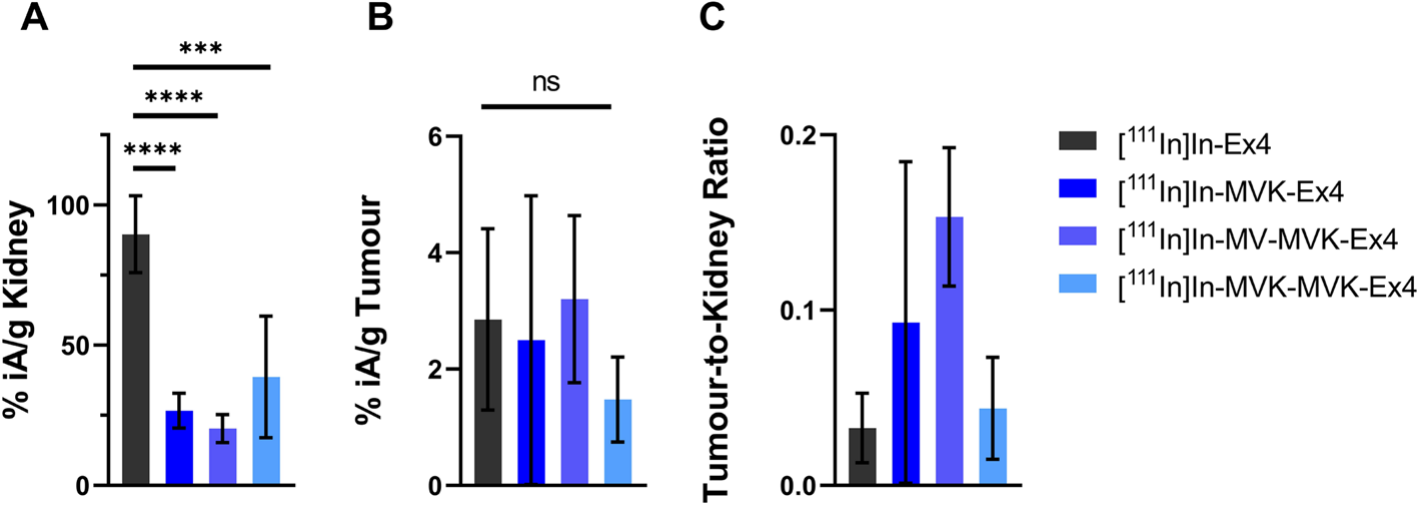
Results from a biodistribution study in CHL GLP-1R tumour-bearing CD1 nu/nu mice 24 h after i.v. administration of the studied exendin derivatives and the reference [^111^In]In-Ex4. (**A**) Kidney uptake 24 h after administration of the [^111^In]In-labelled compounds was significantly reduced in all Ex4 derivatives carrying an MVK cleavable linker. Highest reduction was achieved with [^111^In]In-MV-MVK Ex4, which displayed a 77% lower kidney uptake compared to [^111^In]In-Ex4. (**B**) Tumour uptake of [^111^In]In-Ex4 and its derivatives 24 h after administration. No significant difference in tumour uptake was observed for the different compounds, indicating that the introduction of the cleavable linkers did not affect the in vivo affinity to the target receptor. (**C**) Tumour-to-kidney ratio of all [^111^In]In-Ex4 derivatives. [^111^In]In-MV-MVK-Ex4 displays the highest tumour-to-kidney ratio, slightly outperforming [^111^In]In-MVK-Ex4 with only one cleavable sequence. All tumour-to-kidney ratios of the cleavable linker derivatives [^111^In]In-MVK-Ex4, [^111^In]In-MV-MVK-Ex4 and [^111^In]In-MVK-MVK-Ex4 are higher than that of the reference [^111^In]In-Ex4, with the difference between [^111^In]In-Ex4 and [^111^In]In-MV-MVK-Ex4 being statistically significant. Statistics were conducted using one-way ANOVA with Dunnett’s multiple comparison test (with *p < 0.05, **p < 0.01, ***p < 0.0005 and ****p < 0.0001)

A biodistribution of all radiolabelled Ex4 derivatives was conducted in mice carrying subcutaneous CHL GLP-1R positive tumours. Results showed significantly reduced radioactivity retention in the kidneys for all cleavable linker derivatives 24 h after intravenous injection compared to the reference [^111^In]In-Ex4 (Fig. 4A). Numerically, a reduction of kidney uptake by 70%, 77% and 57% was determined for [^111^In]In-MVK-Ex4, [^111^In]In-MV-MVK-Ex4 and [^111^In]In-MVK-MVK-Ex4, respectively. In contrast to the kidney uptake, there was no significant difference in tumour uptake after 24 h, suggesting that the instillation of the cleavable linkers does not affect in vivo activation of the receptor and subsequent internalization into tumour cells (Fig. 4B). Combining kidney uptake and tumour uptake into the tumour-to-kidney ratio, shows that [^111^In]In-MVK-Ex4, [^111^In]In-MV-MVK-Ex4 and [^111^In]In-MVK-MVK-Ex4 displayed higher ratios than the reference [^111^In]In-Ex4 (Fig. 4C). Indeed, in agreement with our previous findings the highest tumour-to-kidney ratio was achieved with [^111^In]In-MV-MVK-Ex4 carrying two cleavable sequences.

## Discussion

Peptidic radiotracers often suffer from high persistent retention of radioactivity in the kidneys. An example thereof is Ex-4, an incretin mimetic drug that binds to the GLP-1R overexpressed on insulinomas and in hyperinsulinism. Based on the previous successful reduction of kidney uptake in conjunction with FnBPA5.1, we studied the potential of the MVK variants MV-MVK and MVK-MVK to reduce the kidney uptake of radiolabelled Ex4. After successful synthesis, we tested the stability of the radiolabelled Ex4 and its derivatives in plasma. While the reference compound [^111^In]In-Ex4 displayed high stability with 80% intact tracer after 24 h, Ex4 derivatives equipped with a cleavable linker quickly displayed the generation of an unidentified secondary peak in the HPLC chromatogram. Based on previous accounts (Murce et al. 2023; Bendre et al. 2020), we speculated that the observed secondary peak is caused by the oxidation of methionine. Though the generation of the sulfoxide M(O)VK has been shown to impair the recognition by neprilysin, Bendre et al. (2020) found that it still resulted in a significant reduction of the kidney retention, in fact even higher than the one obtained with the non-oxidized version.

Bringing the focus on the kidney cleavage in vivo, the conducted biodistribution showed that the introduction of the cleavable linkers is efficient in reducing the kidney uptake by 70 ± 7% for [^111^In]In-MVK-Ex4, by 77 ± 6% for [^111^In]In-MV-MVK-Ex4 and by 57 ± 24% for [^111^In]In-MVK-MVK-Ex4 at 24 h p.i. Though not significant, the kidney uptake of [^111^In]In-MV-MVK-Ex4 was 7% lower than with the single cleavable linker [^111^In]In-MVK-Ex4. In contrary to [^111^In]In-MV-MVK-Ex4, the dual linker [^111^In]In-MVK-MVK-Ex4 displayed 13% higher kidney retention than [^111^In]In-MVK-Ex4. Even though all cleavable linker derivatives displayed significant reduction of renal radioactivity levels compared to the reference [^111^In]In-Ex4, instillation of a dual MVK sequence did not significantly improve kidney washout compared to the single MVK linker.

Interestingly, in both studies using the MV-MVK and MVK-MVK dual linkers coupled to either FnBPA5.1 or to Ex4, the MV-MVK dual linker, deprived of the Lys, demonstrated the lowest absolute kidney uptake. Data from both [^111^In]In-Ex4 and [^111^In]In-FnBPA5.1 therefore imply, that the Lys inserted between the first linker and the amino-benzyl function in the dual MVK-MVK entity, negatively affects enzymatic breakdown by neprilysin.

To evaluate if the binding of the derivatives to the GLP-1R is hindered by the cleavable linker, we conducted in vitro IC_50_ and cell uptake assays. Despite previous findings by Zhang et al. suggesting that the introduction of MVK between the chelator NOTA and Ex4 does not impair affinity towards the GLP-1R (Zhang et al. 2019), we found that all cleavable linker derivatives displayed lower affinity (higher IC_50_ values) than the reference Ex4. Regardless of the decrease in affinity of [^111^In]In-MVK-Ex4, [^111^In]In-MV-MVK-Ex4 and [^111^In]In-MVK-MVK-Ex4 towards the GLP-1R, cell uptake of the cleavable linker derivatives was only impaired for [^111^In]In-MV-MVK-Ex4, which exhibited a significantly reduced internalized fraction after 2 h. While there is an apparent divergence between affinity and cell uptake, it is important to note that cell uptake not only depends on the affinity of the investigated compound to the receptor, but also on the internalization and recycling rate of the receptor itself. Interestingly, while in vitro data suggested a decreased affinity and for one compound even a decreased cell uptake, there was no difference in tumour uptake between any of the tested compounds in vivo.

Despite the promising further reduction of kidney uptake with the dual cleavable linker MV-MVK in conjunction with FnBPA5.1, our results with Ex4 show that the dual cleavable linker strategy is not per se transferable to all radiotracers suffering from persistent kidney accumulation. In fact, it has been shown earlier that the renal cleavage by neprilysin can be sensitive to changes in the chemical structure of the test compound (Arano 2020) and similarly it is reasonable that the dual MV-MVK linker in conjunction with Ex4 was affected by sterical hindrance. Indeed, while FnBPA5.1 is a naturally unstructured peptide, Ex4 is highly helical in solution (Andersen et al. 2002).

Our previous research as well as literature have shown the potential of cleavable linkers in diminishing persistent kidney retention of radiotracers (Zhang et al. 2019; Valpreda et al. 2022; Vaidyanathan et al. 2018). As shown in this study, in the design of novel cleavable-linker bearing radiopharmaceuticals, it is key to consider and test the linker position, the linker size, enzyme recognition and stability. Apart from technical and methodological pitfalls associated with the use of single or dual MVK linkers in conjunction with radiopharmaceuticals, it will be key to confirm the transferability of the cleavable linker approach in a clinical setting, with human subjects that possess a potentially different metabolism.

## Conclusion

Taken together, our study shows that though the dual MVK cleavable linker approach is advantageous for some targeting moieties, structural characteristics as well as binding modes and the pharmacokinetics of the targeting moiety play a crucial role in determining whether it is efficient or not. Though the dual MV-MVK did not significantly outperform the single MVK linker, it displayed the lowest absolute kidney accumulation and we therefore believe, that the dual approach is valid and when optimized for the corresponding targeting molecule can further decrease radioactive kidney burden. Optimization in terms of length and form of the linking entity between the dual MVK linker and the targeting structure as well as the combination of an MVK sequence with other cleavable linker sequences such as GFK or MWK are conceivable. And finally, a combination of cleavable linkers and albumin binding moieties could also be of interest, especially in the case of Ex4, where albumin binders have already proven successful in decreasing kidney uptake.

## Methods

### General

Ex4 coupled to the chelator 1,4,7-triazacyclononane,1-glutaric acid-4,7-acetic acid (NODAGA) was obtained from piCHEM (Grambach, Austria). Ex4 derivatives MVK-Ex4, MV-MVK-Ex4 and MVK-MVK-Ex4 coupled to NOTA were synthesized in-house with > 95% purity using standard solid-phase peptide synthesis (SPPS). Further information regarding synthesis can be found in the Additional file 1.

### Radiolabelling

Ex4 and its cleavable linker derivatives were radiolabelled with [^111^In]InCl_3_ (Mallinckrodt, Cham, Switzerland) in the presence of 0.5 M ammonium acetate pH 5.5 at a specific activity of 4 MBq/nmol. To prevent oxidation of Met in the radiolabelling solution, D-L-Selenomethionine was added in a 50-fold molar excess to all solutions carrying a Met in their sequence. The radiolabelling mixtures were incubated for 15 min at 50 °C and subjected to reverse phase high pressure liquid chromatography (RP-HPLC, Agilent 1200 Series, Santa Clara, USA) connected to a GinaStar raytest γ-detector (Elysia Raytest, Straubenhardt, Germany). A C18 column (ReproSil-Pur 120, 3 μm, Dr. Maisch GmbH, Germany) with a linear gradient ranging from 95 to 5% of solvent A in 10 min (A: H_2_O containing 0.1% trifluoracetic acid (TFA); B: acetonitrile (MeCN)) was employed.

### Plasma stability

500 μL of human blood plasma (HBP, Blutspendezentrum Kantonsspital Aarau, Switzerland) together with 8 MBq of each radiotracer were incubated at 37 °C with slow agitation. At each time point (0, 1, 4 and 24 h) a sample was drawn and precipitated with a 1:1 mixture of MeCN containing 0.1% TFA and H_2_O. The aliquot was centrifuged for 30 min at 14′000×*g*, filtered and analysed by RP-HPLC using a C4 column (Reprosil Gold 200 3 μm, Dr. Maisch, Ammerbuch, Germany) at a constant flow of 1 mL/min over 30 min starting from 80 to 10% solvent A (H_2_O containing 0.1% TFA) and MeCN as solvent B.

### Cell culture

Stably transfected Chinese hamster lung (CHL) cells expressing the glucagon-like peptide-1 receptor (CHL GLP-1R, a kind gift of Prof. Brigitte Lankat-Buttgereit, Marburg, Germany) were cultured in Dulbecco’s modified Eagle medium (DMEM) containing 4.5 g/L D-glucose. Fetal calf serum (FCS, 10%), 2 mM L-glutamine, 0.5 mg/mL geneticin sulfate, 1 mM sodium pyruvate and 0.1 mM non-essential amino acids and antibiotics (100 IU/mL penicillin, 0.1 mg/mL streptomycin, 0.25 μg/mL fungizone) were added and the cells were maintained under standard conditions at 37 °C in a humidified atmosphere containing 5% CO_2_. Hamster origin of the CHL cell line was confirmed and the cell line was tested negative for mycoplasma (MycoStrip™, InvivoGen).

### Cell uptake

CHL GLP-1R cells were seeded in 6-well plates at a density of 0.75 × 10^6^ cells per well and were grown overnight to reach a confluence of 90–95%. 200 pM of radiolabelled peptide in DMEM containing 0.1% bovine serum albumin (BSA) were added to the cells and incubated at 37 °C for various time points (5, 15, 30, 60 and 120 min). The supernatant was collected and the cells were washed twice with phosphate-buffered saline (PBS). The surface-bound fraction was collected from the cells after incubation with glycine buffer pH 2.8 at room temperature. Finally, cells were incubated in 1 M sodium hydroxide and collected as the internalized fraction. The activity of the samples was measured in the γ-counter (Packard Cobra II Auto Gamma, PerkinElmer, Switzerland). Significance of the obtained data was assessed in Prism (version 7.00, GraphPad Software, La Jolla, USA) using ordinary One-way ANOVA with Dunnett’s multiple comparison test.

### IC_50_ determination

CHL GLP-1R cells were seeded on 12-well plates at a density of 0.2 × 10^6^ cells per well and were grown overnight to reach a confluence of 90–95%. Cells were washed with ice-cold PBS and incubated for 1 h on ice with 200 pM of [^111^In]In-labelled Ex4 supplemented with the corresponding displacer compound (10^−5^ to 10^−10^ M) labelled with [^nat^In]InCl_3_. For cold labelling of the Ex4 derivatives, a two-fold molar excess of [^nat^In]InCl_3_ was added to the corresponding stock solution before dilution into the working concentration using PBS. After incubation, cell supernatant was collected and cells were solubilized with 1 M NaOH. Activity of the collected fractions was measured using a γ-counter. The IC_50_ values were determined by fitting the data with non-linear regression using the least-squares fit of GraphPad Prism. All experiments were performed in triplicates.

### Biodistribution

Female CD1 nu/nu mice (Charles River Laboratories, Sulzfeld, Germany) were inoculated subcutaneously into both shoulders with 8 × 10^6^ CHL GLP-1R cells. After 9 days of tumour growth the animals were injected with 150 kBq (38 pmol) of the corresponding [^111^In]In-labelled Ex4 compounds in 100 μL PBS i.v. into the tail vein. After 24 h mice were euthanized using CO_2_ asphyxiation and organs were collected. Consequently, organs were harvested, weighed and radioactivity measured using a gamma counter. Employing 100 μL of the injected solution as a standard, the percentage of injected activity per gram organ (% iA/g) was calculated. Statistics were performed using OneWay ANOVA with Dunnett’s multiple comparison test in GraphPad Prism.

### Supplementary Information

**Additional file 1.** 1. Chemical Synthesis of Ex4 Cleavable Linker Derivatives, 2. Radiolabelling, 3. STR Profile of Chinese Hamster Lung Cell Line (CHL-GLP-1R), 4. Biodistribution Data, 5. References.

## Data Availability

The datasets analysed during the current study are available from the corresponding author on reasonable request.

## Abbreviations

BSA: Bovine serum albumin
CHI: Congenital hyperinsulinism
CHL: Chinese hamster lung cells
CHL GLP-1R: Chinese hamster lung cells overexpressing the glucagon-like peptide-1 receptor
DMEM: Dulbecco’s modified Eagle medium
Ex4: Exendin-4
FCS: Fetal calf serum
GLP-1R: Glucagon-like peptide-1 receptor
HBP: Human blood plasma
i.v.: Intravenous
NODAGA: 2-[1,4,7-Triazacyclononan-1-yl-4,7-bis(tBu-ester)]-1,5-pentanedioic acid
NOTA: 1,4,7-Triazacyclononane-1,4,7-triacetic acid
PBS: Phosphate-buffered saline
RP-HPLC: Reverse-phase high performance liquid chromatography
PRRT: Peptide receptor radionuclide therapy
TFA: Trifluoroacetic acid
