# Supplementary material for: Reducing kidney uptake of radiolabelled exendin-4 using variants of the renally cleavable linker MVK
Source: EJNMMI Radiopharm Chem. 2023 Sep 4;8:21. doi: 10.1186/s41181-023-00206-2 (PMC10477158; doi:10.1186/s41181-023-00206-2)
Supplement: Supplementary file 1 — Additional file 1. 1. Chemical Synthesis of Ex4 Cleavable Linker Derivatives, 2. Radiolabelling, 3. STR Profile of Chinese Hamster Lung Cell Line (CHL-GLP-1R), 4. Biodistribution Data, 5. References. [file 41181_2023_206_MOESM1_ESM.docx]

**Supporting Information**

# Reducing Kidney Uptake of Radiolabelled Exendin-4 Using Variants of the Renally Cleavable Linker MVK

Belinda Trachsel ^†, §^, Giulia Valpreda ^†, §^, Alexandra Lutz ^§^, Roger Schibli ^†, §^, Linjing Mu ^§^, Martin Béhé^†^

^†^ Center for Radiopharmaceutical Sciences, ETH-PSI-USZ, Paul Scherrer Institute, 5232 Villigen PSI, Switzerland

^§^ Department of Chemistry and Applied Biosciences, Institute of Pharmaceutical Sciences, ETH Zurich, 8093 Zurich, Switzerland

**Table of Contents**

1. **Chemical Synthesis of Ex4 Cleavable Linker Derivatives**

## 1.1 General Procedure for Nle14,Cys40-Ex4 Conjugation via Maleimide-Thiol Chemistry.

## Synthesis of Nle14,Cys40(NOTA-Bn-MVK)-Ex4

## Synthesis of Nle14,Cys40(NOTA-Bn-MVK)-Ex4

## 1.4 Synthesis of Nle14,Cys40(NOTA-Bn-MVK(Me)_2_-amBn-MVK)-Ex4 (Ex4-MVK-MVK)

1. **Radiolabelling**
2. **STR Profile of Chinese Hamster Lung Cell Line (CHL-GLP-1R)**
3. **Biodistribution Data**
4. **References**
5. **Chemical Synthesis of Ex4 Cleavable Linker Derivatives**

Nle14,Lys40-Ex4 (Figure S1) coupled to a NODAGA chelator was purchased from Pichem (Grambach, Austria) and served as the reference compound. Nle14,Cys40-Ex4, the starting material for the attachment of the cleavable linkers was also purchased from Pichem and employed without further purification. All cleavable linkers were synthesized via SPPS as NOTA-based maleimide-activated derivatives employing a standard Fmoc/tBu based approach as described in previous literature. (1) Ex4 derivatives equipped with cleavable linkers were conjugated to Nle14,Cys40-Ex4 via maleimide-thiol chemistry.

***Figure S1.*** *The molecular composition of the reference peptide Ex4 coupled to a NODAGA chelator via a lysine in the paper referred to as Ex4.*

## General Procedure for Nle14,Cys40-Ex4 Conjugation via Maleimide-Thiol Chemistry.

An appropriate volume of the corresponding NOTA-MVK-based maleimide-activated linker at a concentration of 1 mg/mL in metal-free PBS (50 µM, pH 6.5) was added to a solution of Nle14,Lys40-Ex4 at a concentration of 6 mg/mL in in metal-free PBS (50 µM, pH 6.5), in a molar ratio of 1:1. The mixture was gently stirred up to 4 h at rt. The conjugation reaction was monitored by means of analytical HPLC and LRMS until the complete consumption of the maleimide-activated linker entity was recorded. The obtained NOTA-conjugated MVK-based Ex4 derivatives were purified by means of semi-preparative RP-HPLC and then proceeded to radiolabelling.

## Synthesis of Nle14,Cys40(NOTA-Bn-MVK)-Ex4

*Nle14,Cys40(NOTA-Bn-MVK)-Ex4 (MVK-Ex4)* was prepared according to the General Procedure starting from NOTA-Bn-MVK(hex-Maleimide)-OH and Nle14,Cys40-Ex4 (500 µL, 3 mg). Following full consumption of the linker, the solution was directly purified via semi-preparative RP-HPLC with a gradient ranging from 30 to 60 % MeCN + 0.1 % TFA in mQ water + 0.1 % TFA over 15 min (t_r_ = 11.8 min) and obtained in 97 % purity. The isolated yield was 67 % (2.2 mg). ESI-Qq-TOF-MS: m/z calculated [M+4H]^4+^ for Nle14,Cys40(NOTA-Bn-MVK)-Ex4 C_234_H_362_N_60_O_74_S_3_: 1323.1387; m/z found: 1323.1398.

R = Nle14-Ex4

***Figure S2.*** *Molecular composition of Nle14,Cys40(NOTA-Bn-MVK)-Ex4 referred to as MVK-Ex4.*

## Synthesis of Nle14,Cys40(NOTA-Bn-MV-amBn-MVK)-Ex4

Nle14,Cys40(NOTA-Bn-MV-amBn-MVK)-Ex4 was prepared according to the General Procedure starting from NOTA-Bn-MV-amBn-MVK(hex-Maleimide)-OH and Nle14,Cys40-Ex4 (500 µL, 3 mg). Following full consumption of the linker, the solution was directly purified via semi-preparative RP-HPLC with a gradient ranging from 35 to 45 % MeCN + 0.1 % TFA in mQ water + 0.1 % TFA over 30 min (t_r_ = 18.2 min) and obtained in >99 % purity. The isolated yield was 30 % (1.2 mg). MALDI-FTICR-MS: m/z calculated [M+H]^+^ for Nle14,Cys40(NOTA-Bn-MV-amBn-MVK)-Ex4 C_253_H_386_N_63_O_77_S_4_: 5666.7103; m/z found: 5666.7171.

R = Nle14-Ex4

***Figure S3.*** *Molecular composition of Nle14,Cys40(NOTA-Bn-MV-amBn-MVK)-Ex4 referred to as MV-MVK-Ex4.*

## Synthesis of Nle14,Cys40(NOTA-Bn-MVK(Me)_2_-amBn-MVK)-Ex4

Nle14,Cys40(NOTA-Bn-MVK(Me)_2_-amBn-MVK)-Ex4 was prepared according to the General Procedure starting from NOTA-Bn-MVK(Me)_2_-amBn-MVK(hex-Maleimide)-OH and Nle14,Cys40-Ex4 (500 µL, 3 mg). Following full consumption of the linker, the solution was directly purified via semi-preparative RP-HPLC with a gradient ranging from 36 to 44 % MeCN + 0.1 % TFA in mQ water + 0.1 % TFA over 30 min (t_r_ = 13.4 min) and obtained in 98 % purity. The isolated yield was 36 % (1.4 mg). MALDI-FTICR-MS: m/z calculated [M+5H]^5+^ for Nle14,Cys40(NOTA-Bn-MVK(Me)_2_-amBn-MVK)-Ex4 C_261_H_406_N_65_O_78_S_4_: 1165.3731; m/z found: 1165.3726.

R = Nle14-Ex4

***Figure S4.*** *Molecular composition of Nle14,Cys40(NOTA-Bn-MVK(Me)_2_-amBn-MVK)-Ex4 referred to as MVK-MVK-Ex4.*

**2. Radiolabelling**


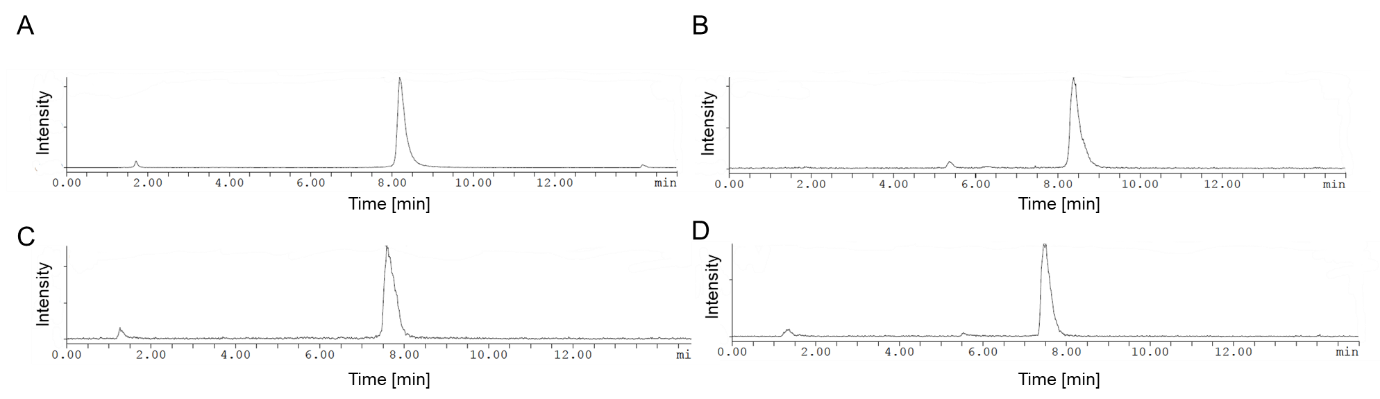


***Figure S5.*** *Typical HPLC radiochromatograms from radiolabellings used for in vitro and in vivo experiments.* ***(A)*** *[^111^In]In-Ex4,* ***(B)*** *[^111^In]In-MVK-Ex4,* ***(C)*** *[^111^In]In-MV-MVK-Ex4,* ***(D)*** *[^111^In]In-MVK-MVK-Ex4.*


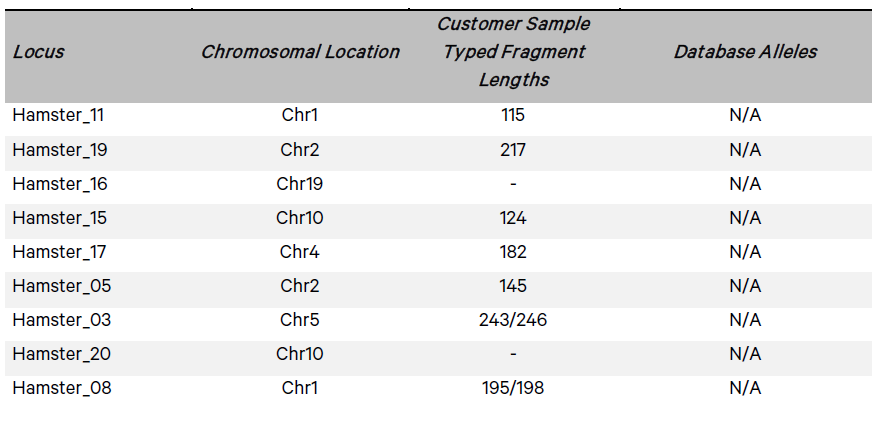
**3. STR Profile of Chinese Hamster Lung Cell Line (CHL-GLP-1R)**

***Figure S6.*** *STR profile of the Chinese hamster cell line transfected with GLP-1R (CHL-GLP-1R). Due to the lack of an official STR databank for hamster cell lines, CHL-GLP-1R cells could not be authenticated. From the STR profile, it is evident, however, that the cell line is of hamster origin.*

**4. Biodistribution Data**

***Table S1.*** *Biodistribution data of ^111^In-labelled Ex4 derivatives in CHL GLP-1R tumor bearing mice at 24 h p.i.*

|  | **[^111^In]In-Ex4** | **[^111^In]In-MVK-Ex4** | **[^111^In]In-MV-MVK-Ex4** | **[^111^In]In-MVK-MVK-Ex4** |
| --- | --- | --- | --- | --- |
| Blood | 0.02 ± 0.00 | 0.02 ± 0.00 | 0.07 ± 0.00 | 0.03 ± 0.01 |
| Heart | 0.07 ± 0.01 | 0.08 ± 0.02 | 0.10 ± 0.02 | 0.08 ± 0.03 |
| Lung | 3.24 ± 1.38 | 8.61 ± 1.23 | 4.83 ± 1.58 | 4.88 ± 1.26 |
| Spleen | 0.11 ± 0.05 | 0.09 ± 0.05 | 0.17 ± 0.02 | 0.24 ±0.04 |
| Pancreas | 2.57 ± 0.75 | 1.43 ± 0.07 | 0.82 ± 0.28 | 1.56 ± 0.63 |
| Liver | 0.14 ± 0.05 | 0.19 ± 0.04 | 0.43 ± 0.05 | 1.32 ± 0.62 |
| Stomach | 0.39 ± 0.17 | 0.36 ± 0.31 | 0.48 ± 0.21 | 0.51 ± 0.23 |
| Intestines | 0.23 ± 0.02 | 0.22 ± 0.08 | 0.17 ± 0.06 | 0.21 ± 0.05 |
| Kidneys | 89.57 ± 13.71 | 26.65 ± 6.22 | 20.26 ± 5.01 | 38.73 ± 21.66 |
| Muscle | 0.08 ± 0.03 | 0.03 ± 0.00 | 0.05 ± 0.01 | 0.04 ± 0.01 |
| Bone | 0.15 ± 0.04 | 0.14 ± 0.05 | 0.12 ± 0.04 | 0.14 ± 0.07 |
| Tumor | 2.85 ± 1.56 | 2.50 ± 2.48 | 3.20 ± 1.44 | 1.48 ± 0.73 |

**5. References**

1. Valpreda G, Trachsel B, Schibli R, Mu LJ, Behe M. Reducing the renal retention of In-111-labelled fibronectin-binding peptides through brush border-enzyme sensitive cleavable linkers. Nuclear Medicine and Biology. 2022;108:S34-S5.
